# Criteria for the selection of complementary private health insurance: the experience of a large organisation in Iran

**DOI:** 10.1186/s12913-022-08777-7

**Published:** 2022-11-19

**Authors:** Mohammad Bazyar, Minoo Alipouri Sakha, Vladimir Sergeevich Gordeev, Batool Mousavi, Amir Karmi, Reza Maniei, Saeed Attari, Mohammad Ranjbar

**Affiliations:** 1 Department of Health Management and Economics, Faculty of Health, Ilam University of Medical Sciences, Ilam, Iran; 2 National Center for Health Insurance Research, Tehran, Iran; 3 Department of Health Education and Promotion, Deputy of Health, Iran University of Medical Sciences, Tehran, Iran; 4 Wolfson Institute of Population Health, Queen Mary University of London, London, UK; 5 Department of Infectious Disease Epidemiology, London School of Hygiene & Tropical Medicine, London, UK; 6 Prevention Department, Janbazan Medical and Engineering Research Centre (JMERC), Tehran, Iran; 7 Janbazan Medical and Engineering Research Centre (JMERC), Tehran, Iran; 8 Health policy and management research center, Department of Health Services Management, School of public health, shahid Sadoughi University of Medical Sciences, Yazd, Iran

**Keywords:** Private health insurance, Benefits package, Multi-criteria decision making, Checklist

## Abstract

**Background:**

Expenses related to employee’s health benefit packages are rising. Hence, organisations are looking for complementary health financing arrangements to provide more financial protection for employees. This study aims to develop criteria to choose the most appropriate complementary health insurance company based on the experience of a large organisation in Iran.

**Methods:**

This study was conducted in 2021 in Iran, in the Foundation of Martyrs and Veterans Affairs to find as many applicable criteria as possible. To develop a comprehensive list of criteria, we used triangulation in data sources, including review of relevant national and international documents, in-depth interviews of key informants, focus group discussion, and examining similar but unpublished checklists used by other organisations in Iran. The list of criteria was prioritised during focus group discussions. We used the best-worst method as a multi-criteria decision making method and a qualitative consensus among the key informants to value the importance of each of the finalised criteria.

**Findings:**

Out of 85 criteria, we selected 28 criteria to choose an insurer for implementing complementary private health insurance. The finalised criteria were fell into six domains: (i) Previous experience of the applicants; (ii) Communication with clients; (iii) Financial status; (iv) Health care providers’ network; (v) Technical infrastructure and workforce; (vi) and Process of reviewing claims and reimbursement.

**Conclusion:**

We propose a quantitative decision-making checklist to choose the best complimentary private health insurance provider. We invite colleagues to utilise, adapt, modify, or develop these criteria to suit their organisational needs. This checklist can be applied in any low- and middle-income country where the industry of complementary health insurance is blooming.

**Supplementary Information:**

The online version contains supplementary material available at 10.1186/s12913-022-08777-7.

## Key messages

- A reliable checklist of criteria to select the most appropriate complementary health insurance company (CHIC) in low- and middle-income countries such as Iran is crucial. This checklist is useful especially, where this selection and the final decision may be affected by personal preferences of the decision-makers.
- According to the context of Iran, the following domains are important in choosing an appropriate CHIC: Previous experience of the applicants; Communication with clients; Financial status; Health care providers’ network; Technical infrastructure and workforce; and Process of reviewing claims and reimbursement.
- The purview of "previous experience of the applicants" received the highest weight in choosing the most appropriate CHIC. This can determine if the CHIC has the potential experience, facilities, and resources to cover the specific health needs of the insured and can deal with the high demands of the population around the country.
- The authors believe that this checklist covers the most important factors which determine the performance of complementary health insurance companies. This checklist can be applied by any organization in Iran and by the other countries with the similar health system context to assess CHICs and have the best choice among them.

## Introduction

In many countries, due to shortages of basic health insurance system and governmental health system, private insurance programs have been introduced to increase financial access and provide extra health care services for people, to reduce the financial burden on overstretched public health financing, and to improve the quality and the efficiency in delivering health care services [1]. Additional health coverage can be purchased through private health insurance in addition to specialised insurance for which only specific individuals are eligible. Despite great diversity in types, benefits, and roles of private health insurance, it can have three forms: substitutive, supplementary, and complementary [2]. Substitutive insurance provides the same or similar benefits as statutory health insurance for people who are either excluded from some or all aspects of statutory cover or allowed to choose between statutory and private coverage [3]. It is often taken up voluntarily either by individuals or more frequently by employers on behalf of employees [4]. Supplementary insurance provides coverage for services already included in the primary insurance program and could be used, for example, to gain faster access or a broader choice to the providers. The complementary private health insurance (CHI) is further differentiated into two subcategories: CHI that covers a limited set of interventions or services not included in the basic publicly funded package (e.g., dental care, optometry, physical therapy, chiropractic care, and outpatient drugs) or only partially covered by the state (e.g., statutory user charges) and CHI that covers co-payments imposed by the statutory health care system [5]. Among these three forms of private health insurance explained above, the role of complementary health insurance is common in Iran.

For all organisations, expenses related to the employee health benefits packages are rising, mainly due to increased health care services utilisation [6]. Hence, organisations are looking for complementary health financing arrangements to provide more financial protection for employees against medical care expenditure [7]. In terms of competition, an employer may shop around for cheaper policies and switch from one insurer to another to find a better deal [3]. However, despite the competition, most complementary health insurance companies (CHIC) offer a similar range of services in nature and type. Therefore, organisations usually outsource specific responsibilities while setting up their healthcare plan to competent and experienced professional CHICs. These CHICs provide the necessary complementary health services on behalf of the employer under a service contract to the employees [6].

To protect the interests of the insured and the beneficiaries, the CHICs must be appropriately and prudently managed. The significant underlying problems of CHICs are lack of relevant expertise, lack of honesty, conflict of interest, or participation in inappropriate decisions. Organisations should select the right insurers to carry out private health insurance activities by collecting performance information to protect the insured [1]. The existing studies propose different selection criteria for private health insurance companies; however, these are not all equally applicable in different situations. The insurers are rated mainly based on sufficient predictive power in statistical data, profitability, solvency cover, investment [8], infrastructure, speed and quality of services provided, internet banking facilities, staff professionalism, and reputation [9]. According to a study in India, the following factors affected the choice of customers for insurance companies: computerisation and online transactions, connectivity with the bank, speed and efficiency of transactions, clear communication, availability of premium collection centre, the reputation of CHIC, professionalism and credibility of staff, fast and efficient counter services, ease of opening the account, secure internet banking [9]. The number of providers in the network, fixed administration costs, and aggregate reinsurance trigger are factors the employers believed were significant in selecting competing insurers [6].

### Health system context in Iran and the role of private health insurance

Currently there is a mix of public and private health insurance schemes in Iran. There are three main basic social health insurance schemes including: the Social Security Insurance Organization (SSIO) for employees of the formal private sectors; the Iran Health Insurance Organization (IHIO) for governmental employees, self-employed, rural residents, and the poor; and the Armed Forces Medical Services Insurance Organization for the armed forces. SSIO insured more than 43 million people and the HIO has insured about 32 million people [10–13]. There are about 17 smaller institutional health insurance funds such as Petroleum Industry Health Organization, National Broadcasting Organization, banks etc. which have launched health insurance coverage for their own employees and dependents outside of the main health insurance organizations [14–16].

In public sector, primary health package including health education, vaccination, preventive and screening services, maternal and child health and environmental health are offered by nationwide primary health network mainly free of charge. Social health insurance agencies offer the health insurance benefit package (HIBP) covering the health services delivered by the secondary and tertiary levels of the health system [17].

As the health services coverage and financial support provided by the basic health insurance organisations have not been strong enough in Iran [10, 16], demand for private health insurance has been rising. In recent decades, with the progress of medical knowledge and technology and the inability of basic health insurance organizations, complementary private health insurers have entered the field of health insurance to address the concerns of people and provide optional PHI [18]. Exclusion of certain health services from basic health insurance coverage (particularly tests, medical appliances, transport costs, corrective lenses, dental care and pharmaceuticals) and not covering the considerable gap between public and private medical tariffs are the main reasons for the development of the market for PHI in Iran.

Private sector of health system in Iran plays an important role in health service provision, mainly focuses on secondary and tertiary health care in urban areas. According to the statistics released in 2019, private health sector and complementary health insurance system accounted for 53.64% and 7.62% of total health expenditure respectively [19]. Complementary health insurance has a nearly 22.9% share of Iran's insurance industry portfolio. In recent years, the share of health insurance is over 16% of the total insurance premiums, and after third party insurance, it has the highest share of the Iranian insurance market. According to the most recent data available, 14.8% of the population is covered by a complementary health insurance system [20].

There are currently 24 complementary insurance companies in Iran that operate under the regulations of the Central Insurance of Iran (in Persian it is called *Bime Markazi Iran*). It was founded in 1971 by the Act of Iranian Parliament for the purpose of regulating, expanding, guiding insurance operations in Iran, and for the guidance of the insurance companies, along with the protection of the insured and their beneficiaries as well as to ensure government supervision of such operations. All of complementary insurance companies are private except one which is called Iran Insurance Company [21].

It is not easy for consumers to choose the insurance policy that best meets their needs as they might find it difficult to understand technical expressions and various terms and conditions in insurance contracts. On the other hand, due to high competition among private health insurance companies in Iran, they do their best to win as many contracts as possible. For instance, CHICs may propose low and non-technical premiums to increase their portfolio and gain more market share which in turn may result in failure to fulfil their commitments. Choosing the wrong company could bring challenges for the insured and lead to dissatisfaction among the beneficiaries. As a result, choosing a proper CHIC at a reasonable price is not an easy decision even for employers. Moreover the final decision may not be the optimum one, as personal preferences of decision-makers may affect the final selection of CHICs. So it is necessary to identify and prioritize the most influencing criteria according to the context of the country for comparing and choosing the best CHIC in Iran.

Foundation of Martyrs and Veterans Affairs (FMVA), (the Persian name of FMVA is *Bonyad Shahid va Omoor Isaargaran*) was one the first organizations in Iran which called for developing a applicable tool including the most important criteria to select the most appropriate CHIC. FMVA is a national and governmental organisation that addresses the affairs of veterans and their dependents all over the country. One of the main affairs is providing health care services and health insurance coverage. These services are financed by the government. Currently about 3.2243.593 people are covered by the FMVA. To the authors' best knowledge, studies on checklist criteria to select the most appropriate CHIC for low- and middle-income countries that can be used during the tender process are lacking. Therefore, we seized this opportunity and select FMVA as our study site to identify such criteria.

## Methods

This study was conducted in 2020-2021 in Iran, using the FMVA as a research site. To find as many applicable criteria as possible and to develop a comprehensive list of criteria, we used triangulation in data sources, including review of relevant national and international documents, in-depth interviews with key informants, focus group discussion with the officials of FMVA and looking for similar unpublished checklists used by other organisations in Iran. The use of multiple data collection methods ensured complementarity, triangulation and validation of data.

### Data collection

#### Document review process

To create an exhaustive list of criteria that can be used to evaluate the performance of CHICs and choose the most appropriate one, at the first step of the study, document review was done. We searched for different sources of information, including books, scientific papers, dissertations, reports, and policy documents to extract as much applicable criteria as possible. We also searched for formal reports published by national and international journals and relevant grey literature using websites of international organisations such as the International Association of Insurance Supervisors. We included documents in Persian and English. To elicit relevant Persian documents, Iranian databases Magiran and SID were searched. For documents in English, the international databases were searched (i.e., PubMed, Google Scholar, and ScienceDirect) using a combination of keywords such as private health insurance, complementary/supplementary health insurance, third party administrator, criteria for rating, tender, and outsourcing. We additionally studied the criteria used to choose a third-party administrator for outsourcing in other industries. No time restrictions were applied, and the search was finalised in September 2020. We reviewed the reference lists of the included documents and found other relevant additional documents for the purposes of the study. Studies were screened by title and abstract independently by two researchers [MAS, MB]. Results were discussed and combined and underwent a full-text review.

#### In-depth interviews with key informants

In-depth interviews of key informants followed the document review to explore the criteria influential in selecting appropriate CHIC. Our participants comprised of FMVA’ officials (provincial health affairs deputy of FMVA), head of regional branches of CHIC, experts from other organisations responsible for holding tenders and selecting the best CHIC, Central Insurance of Iran (department for supervising the health coverage of CHIC), and beneficiaries of FMVA. As officials of FMVA were quite familiar with the best key informants regarding the topic, they facilitated the connection with the provincial health affairs deputies of FMVA. The regional FMVA health affairs deputies were chosen from different geographical and socioeconomic regions of Iran to get more diverse and representative viewpoints. We used purposeful sampling with maximum variations [22, 23] to accurately capture the perspectives of all relevant informants that were experienced in tenders and familiar with criteria applicable for the selection process. We also used snowball sampling and asked interviewees to introduce other key informants. All participants consented to participate.

Interviews took place either face to face or via telephone, using an interview guide and a semi-structured form. The semi-structured interview guide was developed based on the primary factors and main areas derived from the literature review. At the start of each interview, we explained the purpose of the study and assured participants of confidentiality and anonymity of the content of the discussions. Interviews started by a general question about what criteria should be taken into account when selecting a CHIC to provide complementary health insurance coverage. We asked interviewees to explain whether they have been satisfied or unsatisfied with the performance of the CHICs and the main reasons behind it. We also questioned them about the important criteria in the following areas including financial issues; interactions with health care providers for providing required heath care services; physical and electronic infrastructures; receiving and reimbursing claims; and managing the objections of beneficiaries. Totally 21 interviews were done. Due to COVID-19, it was impossible to conduct all interviews in person and also some of the regional FMVA health affairs deputies were inaccessible. For these reasons, in eight cases, the interviews were conducted via telephone. With participants' permission, all calls were recorded. Interviews with the officials and employees lasted for about 40 minutes. Interviews with beneficiaries of FMVA lasted for about ten minutes. Other characteristics of interviewees including their age, gender, and years of working experience are presented in Appendix Table 1.

### Similar checklists from other organisations

We also examined similar but not freely available decision-making checklists to guide the tender procedure from other organisations. To get access to these documents, our research team and officials of FMVA contacted staff at the headquarters of CHICs, large organisations in the capital, Central Insurance of Iran, and Iranian Insurers Syndicate. Capitalising on staff experience and knowledge, we managed to identify two similar checklists from Tehran University of Medical Sciences and Iran Insurance Company. Appendix Table 2 shows which criterion has been extracted from which sources of data.

### Focus group discussions

We held five focus group discussions (FGDs) with the officials of FMVA. The first four FGDs lasted for about 3 hours, while the last lasted for about 4 hours excluding the time of resting and entertaining. The purpose of the first two FGDs was mainly to discuss the necessity, meaning, and applicability of the criteria derived from the in-depth interviews, literature review, and similar checklists from other organisations. Discussing the criteria draft helped clarify them and reminded the participants of other relevant criteria. We tried to find any pertinent possible criteria that could influence CHIC performance in providing health insurance coverage for the insured. During the second FGD, a study findings by the FMVA were presented about the health services utilisation and the main complaints and demands by the FMVA beneficiaries. These complaints and requests by the FMVA beneficiaries helped us to identify other important criteria. Participants were mainly the officials of central headquarters of FMVA in the capital, Tehran. The same 10 participants attended five FGDs. There was only a partial overlap between interviewees and FGDs participants.

### Data analysis

All interviews were transcribed verbatim and analysed by one of the authors (MB). Content analysis using the framework method (the main themes included in the interview guide) was used to analyse the data deductively. We welcomed new themes emerging from the data and remained flexible to add new themes to the initial framework inductively. The criteria were extracted using MAXQDA10 (VERBI Software. *MAXQDA10*. Berlin: VERBI Software, 2010). Criteria initially were grouped into themes (domains). The initial domains and criteria were discussed in FGDs with FMVA officials, and amendments were made whenever necessary. Once the main domains and a comprehensive list of criteria were identified, the analysis aimed to determine the optimal selection of criteria by prioritising them.

### Prioritising the criteria

In the third FGD, the complete list of criteria was presented. These criteria were scored for prioritisation by ten key informants from 1 to 10 based on their necessity, importance, availability of data to measure them, and ability to differentiate between the performance of CHICs. Participants comprised of officials of health affairs deputy of FMVA, regional health deputy of FMVA in Tehran, and those who were directly responsible for holding a tender for CHI coverage in the FMVA. Each participant scored criteria individually. In the fourth session, the results of the third session were presented, and the scores were discussed. Criteria with scores higher than the average were chosen for the next steps. In addition, we retained some below average scored criteria. As the research team believed that they had a significant effect on the satisfaction of beneficiaries and health centres. Therefor before finalising, those criteria were discussed once more in the group. This time, the participants were convinced that those criteria should be kept.

### Valuing and suggesting indicators for the finalised criteria

In the fifth FGD, the officials suggested indicators for the criteria selected from the previous FGD. These indicators aim to measure the state of CHIC participating in the tender of FMVA. We also conducted the best-worst method (BWM) as a multi-criteria decision making method (MCDM) to weight the importance of each of the finalised domains and criteria and ranked them. MCDM is appropriate to use where several alternatives (options) need to be evaluated concerning several criteria and find the importance of the criteria. BWM is a pairwise comparison-based method that offers a structured way to make the comparisons. BWM was applied as it has several significant benefits. For instance, identifying the best and the worst criteria before conducting the pairwise comparisons among the criteria makes the pairwise comparisons more effortless and more reliable. It also needs fewer pairwise comparisons, making it less dull for participants [24, 25]. To compare finalized domains and criteria and to determine their weights using BWM, we developed a questionnaire according to the principles of BWM to make pairwise comparisons ready for the participants. One of the authors (MB) explained how BWM works and how they should make pairwise comparisons. Participants filled out the questionnaires which were analysed by an analyser specialising in MCDM. The scores for each criterion extracted from BWM were discussed once again among members of the Tender Committee in the FMVA. Therefor they modified the scores using qualitative consensus among themselves according to the considerations and requirements of FMVA.

## Results

We could find many diverse criteria applicable for assessing the performance of complementary health insurance companies. These diverse criteria were discussed in FGDs. In the FGDs we changed the spelling of the criteria for clarification, merged the criteria with similar meaning or broke some of them into two or several separate criteria whenever it was necessary. Finally 85 criteria were finalized. The comprehensive list of these 85 criteria and the sources from which they were extracted is presented in appendix Table 2. These criteria were categorized in seven domains. These seven domains include (i) Previous experience of the applicants; (ii) Communication with clients; (iii) Financial status; (iv) Health care providers’ network; (v) Technical infrastructure; (vi) Medical workforce; and (vii) Process of reviewing claims and reimbursement.

In the third and fourth FGDs, out of 85 criteria, 32 criteria were selected. Domains of "Technical infrastructure" and "Medical workforce" were merged together. During the phase of suggesting indicators for criteria to compare and score the performance of CHICs, four criteria were further removed as the participants mentioned that currently no reliable data were available to measure them in Iran accurately (See Appendix Table 3). So the final selected list of criteria for presenting in the tender comprises six domains and 28 criteria (Table 1). Table 1 also shows the weight of each domain and criteria.

**Table 1:** The final checklist of criteria to assess the performance of complementary health insurance companies in Iran

	Domains		Criteria	Weight of each domain (weight out of 100)	Weight of each criterion out of 100, according to the results of Best-Worst Method (weight according to the consensus among experts)
1	Previous experience of the applicants	1	Experience of contraction with organizations with similar population size in the last 10 years	24.42	**12.9** (9.29)
		2	Experience of contraction with organizations with similar beneficiaries’ characteristics (age and gender, special medical needs, etc.) in the last 10 years		**8.97** (8.07)
		3	Number of current medical contracts with large organizations (over 10 thousand beneficiaries )		**3.26** (7.06)
2	Communication with clients	1	Satisfaction of previous organisations which have worked with the applicants (CHICs) in the last 3 years (certificates, awards and official certificates obtained by the company)	22.68	**5.82** (4.63)
		2	Customer grievance management system		**9.77** (6.05)
		3	Comprehensive and up-to-date information system (physical or electronic manual, site, SMS, application, IVR) to inform the insured regarding the health care providers under contraction and the benefit package		**5.26** (6.2)
		4	Emphasizing on public health services and health education		**1.82** (5.8)
3	Financial status	1	Financial strength rate in the last year	16.53	**3.65** (4.47)
		2	The amount of registered capital of the applicants (CHICs)		**6.24** (4.58)
		3	Ratio of the amount of compensation paid to the insured to the total premiums received (treatment compensation coefficient)		**5.03** (4.81)
		4	License to provide reinsurance contract		**1.29** (2.66)
4	Health care providers’ network	1	Number of private hospitals under contraction and their distribution over the country	17.89	**4.93** (2.66)
		2	Number of specialist offices under contraction and their distribution over the country		**2.27** (2.45)
		3	Number of pharmacies under contraction and their distribution over the country		**2.35** (2.61)
		4	Number of laboratories under contraction and their distribution over the country		**2.35** (2.61)
		5	Number of radiology centres under contraction and their distribution over the country		**2.35** (2.61)
		6	Number of first class hospitals under contraction and their distribution over the country		**1.31** (2.4)
		7	Number of public and private policlinics (for outpatient health services) under contraction and their distribution over the country		**2.35** (2.55)
5	Technical infrastructure and workforce	1	Number and distribution of active branches all over the country	11.42	**2.59** (2.06)
		2	Having an online system to check the complementary health insurance coverage status of the insured in the contracted medical centres		**1.65** (2.01)
		3	Having an online reporting system to provide financial reports at regular intervals (based on the requests of the FMVA)		**1.37** (1.92)
		4	Having a central organisational structure in the headquarters to analyse, monitor and control health care expenditures (cost management)		**2.24** (1.92)
		5	Adequacy of the number of personnel specialised in reviewing medical records		**0.83** (1.5)
		6	Possibility of establishing an electronic health record		**2.75** (2.01)
	Process of reviewing claims and reimbursement	1	Having an active application to perform all activities related to reviewing claims online and electronically (ability to send reimbursement SMS to the insured person; sending SMS to the insured person informing them about the health care services provided for them; possibility to follow the process of claims review by the insured online; possibility of announcing the amount of claim which is not payable and why; having electronic medical record)	7.38	**2.08** (1.9)
		2	Time duration of reviewing and reimbursing medical records delivered by the contracted medical centres (one month for hospitals and 14 days for outpatient and para clinic centres)		**1.61** (1.78)
		3	Time duration of reviewing and reimbursing medical records delivered by the insured (14 days)		**2.12** (1.69)
		4	Possibility to barter bonds, stocks, property		**1.57** (2.01)
	Total	28			**100**

First domain, “previous experience of the applicants” with the weight of 24.42, received the first rank among other domains. Other domains including communication with clients (weight 22.68); Health care providers’ network (17.89); financial status (16.53); technical infrastructure and workforce (11.42); and process of reviewing claims and reimbursement (7.38) received highest weights respectively. In the fifth FGD, the experts suggested indicators for each criterion to measure the performance of CHICs participating in the tender of complementary health insurance coverage. We explain the indicators here briefly.

As FMVA is a national organization with a large number of beneficiaries distributed all over the country, it is very important to ensure that the selected CHIC has the potential capacity to meet the demands of this large scattered population. To do so, two criteria of experience of contraction with organizations with “similar population size” and “similar features” were proposed. To compare and score the performance of CHICs, experts suggested giving 10 % of the weight of this criterion (9.29) to each CHIC per 100 thousand insured population. It means that if over the last 10 years, a CHIC has had the experience of providing complementary health insurance coverage for an organization with a large population, for instance, 500 thousand beneficiaries, half of the score (9.29/2 = 4.64) should be given to that CHIC, and total score for this criterion is given to those companies with experience of providing complementary health insurance coverage for 1 million people or more. Companies with contracts less than 1 hundred population in the past will not get any score for this criterion. On the other hand, a part of FMVA’ beneficiaries composed of veterans and injured people in wars suffering from chronic diseases like respiratory disorders. This is the reason for using health care services much more than an average person. Experts from FMVA suggested that those CHICs, which have the experience of working with organizations, where a significant part of their population made up of elderly or suffering from chronic diseases, probably have the potentiality to meet the health needs of FMVA’ beneficiaries. Most of CHICs might not have worked for large population, but they might have provided complementary health insurance coverage for many organizations with smaller populations successively. To take into account this important factor (weight 7.06), experts assigned 10 % of the score per each contract with organizations over 10 thousand insured population. That means if a CHIC presents 10 contracts, each more than 10 thousand people, it is given the total score 7.06 or of it provides 5 contracts, it will get 3.53 score for this criteria.

The next domain, communication with clients, mainly addresses the satisfaction of organizations and the beneficiaries that have got their complementary health insurance coverage from those CHICs. As there is no formal and reliable system for surveying the satisfaction of the insured regarding the quality of CHICs’ performance in Iran, experts suggested providing formal certificated or documents by the CHICs to show the satisfaction of organizations they have provided complementary health insurance coverage for them. Experts proposed 10% of the score for each certificate provided by the CHICs. For the criterion “customer grievance management system” experts suggested giving 25% of the score (6.05) for each of the following systems including grievance management office, grievance record system, Interactive voice response (IVR), and grievance management application. To assess the status of CHICs in terms of having a comprehensive and up-to-date information system to inform the insured regarding the health care providers’ network and the health services included in the benefit package, experts suggested to give 20 % of score for each of following facilities such as physical or electronic manual, online site, SMS, application, and IVR. If a CHIC provides all of these five facilities, it is given full score. Currently preventive and public health services are provided for Iranians by the primary level of health system “district health network” free of charge and basic and complementary health insurance organizations covers mainly health services provided in the secondary and tertiary levels of the health system. Despite this, FMVA’ officials insisted on putting a criterion addressing public health and health education activities as a way to manage health insurance premiums. To assess how public health oriented the CHICs are, experts suggested giving 25% of the score (5.8) for each of the following public health activities if they are provided by the CHICs: holding conferences, preparing pamphlets, holding training workshops, and preparing training videos about public health issues for the insured.

The domain of “financial status of CHICs” composes of three criteria. First criterion is financial strength rate in the last year ranged from 1 (good) to 3 (not good). The strength of CHICs is determined by the Central Insurance Organization. Total score of this criterion is given to the companies with rate 1, 75 % of score for companies with rate 2, and no score for companies with lower rates. As this criterion does not assess the volume of financial resources and properties of CHICs, the second criterion address the amount of registered capital of the CHICs directly. Experts did not propose minimum amount of capital for assessing the CHICs, instead they suggested to sort the CHICs descending based on their capital, and then 100 % of score for the first company, the rest of the companies get scored proportionally compared to the first company. Next financial criterion, treatment compensation coefficient, means that whether premiums collected would cover the cost of predicted health services used by the insured or not. Experts suggested that the optimal range of compensation coefficient (premiums divided by health care expenditures) is 75 to 85 %. The ratio higher than that means that the CHICs could not manage their financial stability and they will not afford reimbursing the health expenditures of the insured which in turn will lead to dissatisfaction among the beneficiaries. On the other hand, lower rations may indicate that CHICs are so strict in reimbursing and focus on their own profit than reviewing claims and reimbursing them. Experts suggested following scoring for this criterion: total score (4.81) for companies with optimal range of compensation coefficient (75 to 85 %) and 10 % reduction in the score per 5% difference (upper or lower than the optimal coefficient range). For the last financial criterion, total score is given to those CHICs which have the license to provide reinsurance contract and no score for companies with no license.

To compare the status of CHICs in terms of adequacy of “health care providers’ network”, experts proposed 7 indicators to cover the following health centres including: private hospitals, offices of specialists, pharmacies, laboratories, radiology, first class hospitals and public and private policlinics. Experts suggested the scoring of the CHICs for this domain according to adequacy of health care providers’ network for their previous contracts. For instance total score (2.66) is given to those CHICs that have contracted at least one private hospital per 2500 insured persons. They proposed to reduce 20 % of score per 500 insured persons increase in ratio of insured persons/one private hospital. This formula was proposed for laboratories, radiology, first class hospitals and public and private policlinics. For specialist offices and pharmacies, ration of 1200 insured persons per center was proposed.

To deal with the needs of FMVA’ beneficiaries all over the country, experts insisted that CHICs should have at least one active branch in each province. Iran is composed of 33 provinces, because of that experts proposed giving 1/33 of score for having one active branch in each province. Health centers should be able to check online whether the insured is covered or not. Most of CHICs have this possibility. Experts weighted this criterion 2.01 and this score is given to any CHIC which proves having this online system. Also FMVA’ officials stated that to monitor the performance of winner of the tender, they may need requesting the winner to provide different financial reports about the health expenditures of the beneficiaries and also expect CHICs to have a central organisational structure for analysing, monitoring and controlling health care expenditures (cost management). CHICs with such a structure and online system to provide any financial report upon request will be given total score and those CHICs not having such systems get no score. The next criterion was adequate number of personnel specialised in reviewing medical records. The weight of this criterion was 1.5 and total score is given to those CHICs which has 1 employee for every 1000 insured populations. Every 100 insured persons increase in per capita reduces the score by 10 %. Electronic health record in Iran is at early stages and CHICs are not still connected to this system. As it was important for FMVA’ officials to move towards electronic health records, they proposed 2.01 score for CHICs with such a possibility.

The last domain was about the process and speed of reviewing and reimbursing claims. Having an active application or system to perform all activities related to claim review online was weighted 1.9. This score was divided into 4 sections and 25% of the score is given for having each of the following possibilities: ability to send reimbursement SMS to the insured; sending SMS to the insured informing them about the health care services provided for them; possibility to follow the process of claims review by the insured online; and possibility of announcing the amount of claim which is not payable and why. The usual time period for reviewing and reimbursing medical records delivered by the medical centres is one month for hospitals and 14 days for outpatient and para-clinic centres and those medical records delivered by the insured themselves. Total score is given to the CHICs that review and reimburse the claims within the above time periods and 35 % of score is reduced for each month of delay for hospital claims and 10 % reduction in score for each week of delay for outpatient claims.

## Discussion

Providing consumers with a broader choice of insurance products and increasing competition among insurance companies is one of the ultimate objectives of insurance markets [3]. We aimed to present a list of influential criteria important in choosing the best CHIC among competing companies in Iran. We organized the criteria in seven domains. The domains of "previous experience of the applicants" and "process of reviewing claims and reimbursement" received the highest and lowest weight respectively.

The first domain of criteria is "previous experience of the CHICs". As FMVA is a national organization with large number of beneficiaries distributed all over the country, it is very important to choose the right CHIC to provide services for the beneficiaries with the least challenges as possible. This sphere assures that the CHIC has the necessary experience, facilities, and resources to cover the specific health needs of the FMVA population and can deal with the high demands of the population around the country. Similar to our findings, in Germany, the supervisory body only permits insurers who specialise in health insurance to operate in the field of voluntary health insurance business to protect policyholders from insolvency arising from other companies [3].

"Communication with clients" is the second domain that ensures the availability of facilities, the ability to address the demands and needs of the insured and provide health services as smoothly and efficiently as possible. Beneficiaries must have access to clear and sufficient information on the price, quality and conditions of CHI products. This enables informed choices about the most suitable CHI product. In line with our findings, the competition watchdog in the United Kingdom (Office of Fair Trading) recommended that insurers develop a transparency code in hospital selection procedures, implying that subscribers should be fully informed of their rights to receive treatment from particular hospitals or hospitals consultant. Subscribers in the Netherlands have access to information about prices and policy conditions in substitutive voluntary health insurance [26, 27]. A study by Mathur et al. (2014) showed that clear communication and cell phone banking facilities influence customers' choice of insurance company [9]. The satisfied consumer perception matters greatly since it directly impacts the customer's confidence in purchasing a health insurance policy. The health insurance company's past performance and reputation among the public can also boost customer perception. Blodgett et al. (1997) stated that customers prefer to purchase health insurance policies based on the recommendations from their friends and relatives through word of mouth [7]. Even though data regarding the satisfaction of different groups of the insured with the performance of their CHICs is important, unfortunately, there is a lack of reliable recorded data in Iran to help decision-makers select the best CHIC more consciously. To fill this gap, the participants suggested using the satisfaction of the top managers of previous organisations contracted with the CHICs in the last three years. It is recommended that to introduce Consumer Assessment of Healthcare Providers and Systems (CAHPS) surveys in Iran ask patients regularly to report on their experiences with a range of healthcare services at multiple levels of the delivery system.

"The financial stability" and financial strength of CHICs, the third domain, can guarantee that they would fulfil the obligations as the commercial insurers' existence depends on the sufficiency of the coverage of the insurer's administrative costs and expected profits. We selected several different financial criteria that can measure various financial stability aspects. Financial strength rate and treatment compensation coefficient were among financial criteria that show how well each CHICs performs financially and whether it is profitable regardless of its size or not. We added two other financial criteria to evaluate the volume of financial assets. These two criteria were the amount of registered financial assets of the CHICs and the license to provide a reinsurance contract. Small companies with a low volume of financial assets might not fulfil their obligations for a large population regardless of how profitable they are. As the FMVA is a governmental publicly funded organisation, the officials of FMVA were additionally concerned that the CHICs should be financially strong enough to continue with their obligations, even if government funding is delayed for whatever reason. Financial regulation is concerned with ensuring that the insurer remains solvent. In the United Kingdom, the supervisory authority's role is to examine detailed financial returns on business [3].

At a minimum, a regulatory entity will require financial information from insurers regarding their reserves, risk categories of their investments, and cash flow. Data on utilisation patterns, enrolment, claims experience, and administrative costs are also essential and can be used to forecast whether an insurance company might be at risk for failure so that early actions can be taken. Health services information is also required and includes provider lists, licences and accreditation certificates to ensure quality and the locations of all providers to verify geographic access [19]. An approach that policymakers can use in developing a regulatory scheme for CHICs has been proposed by Sekhri (2008) and consists of addressing five key questions on interactions between key actors in the health insurance market: the insurers, the consumers, and the providers. The first question is about the seller of insurance. He believes that only appropriate institutions with financial means and possess adequate human and technical resources to provide optimal services to users can sell insurance [20].

The fourth domain in selecting CHICs is the number of health care providers and health centres under contraction. The adequate number of different kinds of providers and health centres is essential to provide required health services for the beneficiaries timely and at the nearest location possible. It increases the right of beneficiaries to choose from a broader range of providers according to their quality and cost, vicinity and accessibility. The intention to purchase a health insurance policy is, thus, significantly and positively related to the quality of the health services and connected to the referral hospitals' standard. It can also have a significant effect on the satisfaction of the insured. Although many contracted providers are beneficial to the beneficiaries, a minimum acceptable number of providers and their distribution are also essential. Participants suggested the minimum acceptable per capita ratio of the number of providers per the insured for each province to quantify these factors. The number of medical centres and health care providers that have cancelled their contracts over the last few years due to the poor performance of CHICs can also be a suitable criterion that shows the level of satisfaction with CHICs. However, participants did not choose this factor as they believed that currently there is no reliable data or an easy way to measure it in Iran. Over the last years, the Ministry of Health and Medical Education in Iran allowed public hospitals to furnish 10 % of their hospital beds for VIP purposes (these wards have higher standards for health provision, for instance, they should be separated from other parts of the hospital, luxurious single bedrooms with private facilities for the patients, food menu, etc.). The main target of these VIP wards is the market of complementary health insurance. Admitting the beneficiaries of FMVA in VIP wards can increase their satisfaction, especially in deprived areas, where the private sector is not available, although it increases the cost of CHICs. Although FMVA’ patients may use VIP wards in state hospitals, however, officials of FMVA did not choose this item as a criteria in the tender of choosing CHICs to control the health care expenditure of FMVA.

The fifth domain was “technical infrastructure and workforce”. The technical infrastructure and workforce of CHICs can accelerate the speed and smoothness of operational activities. Having more active branches all over the country makes it easier to handle and respond to the demands of the beneficiaries. According to the participants having at least one active branch in each province was necessary, and each province was given 1/33 of the total score for this criterion. Adequate number of personnel specialised in reviewing medical records increases the speed of claim review processing. Also, some of the interviewees emphasised that the CHICs should have their own personnel for reviewing medical claims and do not outsource the process of reviewing claims to other agencies. They believed when the CHICs have their own personnel for review of claims, it makes a claim check much easier and faster. They can also communicate directly with the insured if they have questions or objections about the reimbursements, reducing the number of complaints by the patients.

The last domain was receiving the claims and reimbursing them at the earliest time and easiest way possible. This is particularly important for the claims from non-contracted medical centres as the insured should submit the required documents to the CHICs offices in person. To do so, the insured may face a lot of challenges. They may not know which documents they should submit, so they may have to go back to the CHICs offices several times. This makes the process of reviewing claims and reimbursement more time consuming and bothersome especially for outpatient and ordinary health services, which usually do not cost much. The beneficiaries may not even know when they are reimbursed, why they cannot get the full reimbursement, or why a part of their claims is not payable. These challenges can make the insured quite dissatisfied and lead to complaints. However, an easy-to-use online system for processing medical claims can solve most of these concerns.

In previous studies, various criteria have been used to rate insurers. Insurers are ranked and rated based on cost-effectiveness, sufficient predictive power in statistical data, profitability, solvency cover, investment, and underwriting risk [8]. Other factors for company selection include location, infrastructure, speed and quality of services provided, internet banking facilities, staff professionalism and guidance, and the firm's reputation [9]. The number of providers in the network, network discounts that would affect expected claims, fixed administration costs and aggregate reinsurance trigger are factors that the employers believed were significant in selecting between competing CHICs [6].

Our proposed criteria comprise a list of mandatory criteria for all CHICs participating in the tender by FMVA and could be quantified. Moreover, each CHIC that intends to participate in the tender should pass several requirements, for instance they should get approval from the Central Insurance of Iran to operate. They also should provide financial warranties from the bank to assure to compensate the loss in the case of failure to meet their obligations. Companies that don't have approval or guarantees cannot participate in the tender. Among CHICs in Iran, only one operates just in the health area. Other CHICs provide various policies such as property, casualty, and life alongside health coverage. According to the participants, the health section is not profitable for CHICs in general, so they cover the loss in the health section by the benefits they get by offering other policies. As a result, participants stated that operating exclusively in the health section for the CHICs is considered a disadvantage rather than an advantage in Iran's current situation. We also restricted the number of suggested criteria by eliminating overlap and grouping similar criteria. For example, two criteria of "experience of having contracts with a similar number of the insured population" and "experience of having contracts with similar monetary value" were merged, because contractions of CHICs with organisations having a large population are usually high in monetary value. Hence, we chose only one of them (the former).

We also kept some criteria that are currently not common in CHICs. For instance, District Health Network in Iran provides public and preventive health care services free of charge. Because of that, health insurance organisations, especially CHICs, are not interested in "providing public health services and health education". However, the officials of FMVA insisted on keeping this criterion to signal what is essential for them and make CHICs consider public health services when managing their financial resources. This criterion became necessary during the COVID-19 pandemic, as the officials of CHIC currently under contracts with FMVA have suggested vaccinating the beneficiaries of FMVA against COVID-19 free of charge to reduce paying high expenditures for treating the patients in the hospital.

### Limitations and strengths of the study

To the best of the authors' knowledge, this is one of the first studies in Iran to present a list of the most important criteria to compare the performance of CHICs in Iran and differentiate good companies from bad ones. The authors believe that this checklist covers the most important criteria which determine the performance of complementary health insurance companies. The checklist can be applied by any organization in Iran and other countries with the similar health system context. Even other organizations and countries with different context can find this checklist and comprehensive list of criteria (Table 2 appendix) as an excellent starting point to create a tailored list of criteria for choosing the most appropriate CHIC. But they can adapt and modify the checklist to suit organisational priorities and requirements and be tailored to the country’s specific context.

## Conclusion

We developed the checklist to choose the best CHIC between competing companies to win the tender for providing CHI coverage. The checklist can be so informative and applicable for other organizations as we believe it covers the main criteria and domains which are helpful in assessing the performance of CHICs. Due to the dynamic nature of the CHI industry and health services system in Iran and likely changes in demands of the officials and beneficiaries of FMVA over time, the officials of FMVA may need to modify the checklist and include new criteria or exclude some of the current criteria or change the weight of criteria according to the changes in the environment. They may also add new indicators for measuring the criteria and make them more precise in differentiating the performances of CHICs. However, our findings suggest a way forward for an evidence-based and quantitative way to support decision-making in the tender process of CHI coverage in Iran. Authors believe that other organisations also can utilise, adapt, modify or develop these criteria to suit their organisational needs. This checklist can be applied to other low- and middle-income countries where the industry of CHI is blooming.

## Supplementary Information

**Additional file 1.**

## Data Availability

All raw data and also the file of this study have been prepared in Persian (not English). But the corresponding author will gladly provide any supporting materials upon request.

## Abbreviation

CHI: complementary private health insurance
CHIC: complementary health insurance companies
FMVA: Foundation of Martyrs and Veterans Affairs
FGDs: focus group discussions
BWM: best-worst method
MCDM: multi-criteria decision making method
JMERC: Janbazan Medical and Engineering Research Center
