# Supplementary material for: Criteria for the selection of complementary private health insurance: the experience of a large organisation in Iran
Source: BMC Health Serv Res. 2022 Nov 19;22:1377. doi: 10.1186/s12913-022-08777-7 (PMC9675121; doi:10.1186/s12913-022-08777-7)
Supplement: Supplementary file 1 — Additional file 1. [file 12913_2022_8777_MOESM1_ESM.docx]

**Appendix Table 1: Characteristics of interviewees**

|  | Position | Number of interviewees | Age | Sex | Years of experience |
| --- | --- | --- | --- | --- | --- |
|  | Provincial health affairs deputy of Martyrs and Veterans Affairs Foundation | 6 | 35-50 | 4 males and 2 females | 15-30 |
|  | Head of provincial branches of complementary health insurance companies | 5 | 35-45 | 2 males and 1 females | 10-15 |
|  | Employees working in departments directly related to complementary health insurance affairs | 4 | 30-40 | 3 males and 1 females | 10-15 |
|  | Central Insurance of Iran (department for supervising the health insurance coverage of complementary insurance companies) | 2 | 50 | Male | 23 |
|  | Beneficiaries (patients) of Martyrs and Veterans Affairs Foundation | 4 | 45-50 | 4 males | - |
|  | Total | 21 |  |  |  |

Appendix Table 2: Complete list of criteria to choose an insurer for the implementation of complementary health insurance

|  | **Domains** |  | **Criteria** | **Source of criteria extraction** | | |
| --- | --- | --- | --- | --- | --- | --- |
|  |  |  |  | In-depth Interview and FGDs | Literature review | Similar checklists |
| 1 | Previous experience of the applicants | 1 | Experience of contraction with organizations with similar population size | * | * | * |
|  |  | 2 | Experience of contraction with organizations with similar beneficiaries’ characteristics (age and gender, special medical needs, etc.) | * |  |  |
|  |  | 3 | Experience of contractions with similar monetary value | * | * |  |
|  |  | 4 | Similar experience in terms of paying heavy claims |  | * |  |
|  |  | 5 | Year of establishment (years of operation) of CHICs | * |  | * |
|  |  | 6 | Having consecutive renewal of health insurance contract with the same organizations in the last four years |  |  | * |
|  |  | 7 | Number of health insurance contracts in the last 5 years |  |  | * |
|  |  | 8 | Number of inpatient and outpatient claims reviewed in the last year (number of claims relative to the insured population, rate of accepted and rejected claims) |  | * |  |
|  |  | 9 | Working exclusively in the field of health or working in different fields including health | * |  |  |
| 2 | Communication with clients | 1 | Satisfaction of the insured under the coverage of CHIC in the last year | * |  |  |
|  |  | 2 | Satisfaction of organizations’ officials contracted with CHIC in the last 3 years | * |  |  |
|  |  | 3 | Number of complaints (objections) raised by the insured against the CHIC in the last year |  | * |  |
|  |  | 4 | Comprehensive and up-to-date information system to inform the insured regarding the contracted and non-contracted medical centers |  |  | * |
|  |  | 5 | Comprehensive and up-to-date information system to inform the insured regarding the benefit package under coverage |  |  | * |
|  |  | 6 | The fame and reputation of the insurance company | * |  |  |
|  |  | 7 | Official certificates and awards obtained by the CHICs |  | * |  |
|  |  | 8 | Variety of health insurance services included in benefit package (dentistry, glasses, hearing aids, foreign medicines, speech therapy, complementary/alternative medicine including traditional medicine, herbal medicines, home care services, hydrotherapy, etc.) | * | * |  |
|  |  | 9 | Informing the insured appropriately when issuing the insurance policy |  | * |  |
|  |  | 10 | Providing public and preventive health services for the insured | * |  |  |
|  |  | 11 | Providing health education interventions for the insured | * |  |  |
|  |  | 12 | No experience of completed or on-going fraud case | * | * |  |
|  |  | 13 | Having reliable and up-to-date advertising system |  | * |  |
|  |  | 14 | Providing several benefits packages with different premiums to increase the right of beneficiaries to choose among them | * |  |  |
| 3 | Financial status | 1 | Status of financial strength in the last year | * | * | * |
|  |  | 2 | Amount of liquidity, capital and annual turnover of the insurance company in the last year | * | * |  |
|  |  | 3 | Ratio of the amount of compensation paid to the insured by the total premiums received (treatment compensation coefficient) | * | * |  |
|  |  | 4 | License to provide reinsurance contract |  | * |  |
|  |  | 5 | The amount of tax paid by the insurance company during the last 5 years |  |  | * |
|  |  | 6 | Adequacy of bank guarantees |  | * | * |
|  |  | 7 | Being financially strong enough to continue reimbursing the claims of the insured and health care centres even without receiving premiums (as Martyr and Veterans Affairs Foundation is a governmental organisation and sometimes the premiums may be allocated by the government several months late) | * |  |  |
|  |  | 8 | Ability to pay heavy claims at one time |  | * | * |
|  |  | 9 | Amount of premium proposed by the insurance company to win the tender | * |  |  |
|  |  | 10 | Giving reliable warranty to compensate the losses caused by the CHIC in case of not being able to fulfill the obligations | * |  |  |
|  |  | 11 | Covering and paying the increase in the price of medicines and medical supplies which may occur during the period of contraction | * |  |  |
|  |  | 12 | Covering the new insured during the period of contraction | * |  |  |
|  |  | 13 | Giving an fixed amount of money to the Martyr and Veterans Affairs Foundation for managing daily needs |  |  | * |
|  |  | 14 | Preparing online financial reports for the FMVA at regular intervals basis |  | * |  |
|  |  | 15 | Possibility of investment and generating more money from the health insurance premiums | * |  |  |
|  |  | 16 | Receiving the health insurance premiums monthly (not annually) | * |  |  |
| 4 | Health care providers’ network | 1 | Number of private hospitals under contraction with CHIC and their distribution over the country | * |  | * |
|  |  | 2 | Number of specialist offices under contraction with CHIC and their distribution over the country | * |  |  |
|  |  | 3 | Number of pharmacies under contraction with CHIC and their distribution over the country | * |  |  |
|  |  | 4 | Number of laboratories under contraction with CHIC and their distribution over the country | * |  |  |
|  |  | 5 | Number of radiology under contraction with CHIC and their distribution over the country | * |  |  |
|  |  | 6 | Number of first class hospitals under contraction with CHIC and their distribution over the country | * |  |  |
|  |  | 7 | Number of public and private policlinics under contraction (for outpatient health services) with CHIC and their distribution over the country | * |  | * |
|  |  | 8 | Number of governmental hospitals under contraction (for outpatient health services) with CHIC and their distribution over the country | * |  |  |
|  |  | 9 | Number of dental clinics under contraction with CHIC and their distribution over the country |  |  |  |
|  |  | 10 | Number of medical centres and health care providers that have cancelled their contracts in the last year due to poor performance of CHIC |  |  | * |
|  |  | 11 | Being able to contract with medical centres with lower medical tariffs | * |  |  |
|  |  | 12 | Accepting and reimbursing the claims of patients receiving their health services from other countries | * |  |  |
|  |  | 13 | Admitting the beneficiaries in the VIP wards in the public hospitals | * |  | * |
|  |  | 14 | Satisfaction and trust of health care providers and medical centres with the performance of CHIC | * |  | * |
| 5 | Technical infrastructure | 1 | Number of provincial branches | * | * |  |
|  |  | 2 | Having an online system to check the complementary health insurance coverage status of the insured in the contracted medical centres | * |  | * |
|  |  | 3 | Having a central organizational structure to analyze, monitor and control health care expenditures (cost management) |  | * |  |
|  |  | 4 | Having an specialized application to smooth insurance operations | * |  |  |
|  |  | 5 | Possibility of establishing an electronic health record | * |  |  |
|  |  | 6 | Having a formal license from the Central Insurance Organization to operate as a complementary insurance company | * |  | * |
|  |  | 7 | Having IVR for addressing customers complaints | * |  | * |
|  |  | 8 | The speed of addressing the customers complaints | * |  |  |
|  |  | 9 | Infrastructure to control medical frauds | * |  |  |
|  |  | 10 | The time needed to import the information of the insured population in the electronic system of insurance company |  | * |  |
|  |  | 11 | Administrative cost (efficiency in implementing operational processes) |  |  |  |
| 6 | Medical workforce | 1 | Number of insurance manpower expert in reviewing medical records | * |  | * |
|  |  | 2 | Years of work experience of manpower in the field of reviewing medical claims | * |  | * |
|  |  | 3 | Having a separate department for the medical affairs in the insurance company |  |  | * |
|  | Process of reviewing claims and reimbursement | 1 | Process of reviewing claims of patients receiving their health services from non-contracted medical centers | * |  |  |
|  |  | 2 | Process of reviewing claims of patients living in remote areas | * |  |  |
|  |  | 3 | Electronic system to allow patients to follow the process of reviewing and reimbursing their claims | * |  |  |
|  |  | 4 | Right of reviewing of medical claims by the experts of the Martyr and Veterans Affairs Foundation if necessary | * |  |  |
|  |  | 5 | Reimbursing the medical claims completely if patients receive their health care services from non-contracted medical centres (as a penalty for not contracting with the vast majority of health care providers) | * |  |  |
|  |  | 6 | Rate of reimbursement (what percentage of medical claims is reimbursed by the CHICs, as one of the common objections of the insured is that CHICs do not reimburse claims for no acceptable reasons or excuses) | * |  |  |
|  |  | 7 | Reimbursing the medical claims according to the cost paid by the patient not based on the medical tariffs | * |  |  |
|  |  | 8 | Autonomy of regional or provincial branches of insurance company for paying medical claims (no need to get permission for everything or expensive claims from the central headquarters) | * |  |  |
|  |  | 9 | Notifying the insured about the reason and amount of the medical claim which is not covered by the insurance (by sending SMS or launching an online system) | * |  |  |
|  |  | 10 | Notifying the insured about the documents required for reimbursing their medical claims | * |  |  |
|  |  | 11 | Sending notification via SMS to inform beneficiaries when their medical claims is reimbursed | * |  |  |
|  |  | 12 | Sending notification (such as SMS) to the beneficiaries to inform them what kind of health services have been provided for them by the medical centres for the patients when recording the medical claims (effective in recognizing the probable frauds committed by the medical centres) | * |  |  |
|  |  | 13 | Time opened for receiving medical claims and post-contract reimbursement |  | * |  |
|  |  | 14 | The time it takes to review and reimburse medical records delivered by the medical centres (one month for hospitals and 14 days for outpatient and Para clinic centres ) | * |  | * |
|  |  | 15 | The time it takes to review and reimburse the medical claims delivered by the insured (for getting their health services from non-contracted medical centres) 14 days | * |  | * |
|  |  | 16 | Different time periods for reviewing and reimbursing medical records based on their cost (paying the less expensive claims sooner) | * |  |  |
|  |  | 17 | Possibility to barter bonds, stocks, property | * |  |  |
|  |  | 18 | Access of the Martyr and Veterans Affairs Foundation' experts to the online system of insurance company to review and evaluate the process of review claims if necessary | * |  |  |
|  | Total | 85 |  |  |  |  |

Note: * - each criterion has been mentioned by which sources.

**Appendix Table 3: Criteria which were deleted in the phase of suggesting indicators**

| **1** | Being financially strong enough to continue reimbursing the claims of the insured and health care centres even without receiving premiums (as FMVA is a governmental organisation and sometimes the budget may not be allocated for several months) |
| --- | --- |
| **2** | The time needed to import the information of the insured population in the online system of insurance company |
| **3** | Time opened for receiving medical claims and post-contract reimbursement |
| **4** | Number of medical centres and health care providers that have cancelled their contracts in the last year due to poor performance of CHIC |
